# Species richness and phylogenetic diversity of seed plants across vegetation zones of Mount Kenya, East Africa

**DOI:** 10.1002/ece3.4428

**Published:** 2018-08-13

**Authors:** Yadong Zhou, Sichong Chen, Guangwan Hu, Geoffrey Mwachala, Xue Yan, Qingfeng Wang

**Affiliations:** ^1^ Wuhan Botanical Garden Chinese Academy of Sciences Wuhan China; ^2^ Sino‐Africa Joint Research Center Chinese Academy of Sciences Wuhan China; ^3^ Mitrani Department of Desert Ecology Swiss Institute for Dryland Environmental and Energy Research Jacob Blaustein Institutes for Desert Research Ben‐Gurion University of the Negev Midreshet Ben‐Gurion Beer‐Sheva Israel; ^4^ East African Herbarium National Museums of Kenya Nairobi Kenya

**Keywords:** climate change, Eastern Afro‐montane biodiversity, montane vegetation, phylogenetic structure

## Abstract

Mount Kenya is of ecological importance in tropical east Africa due to the dramatic gradient in vegetation types that can be observed from low to high elevation zones. However, species richness and phylogenetic diversity of this mountain have not been well studied. Here, we surveyed distribution patterns for a total of 1,335 seed plants of this mountain and calculated species richness and phylogenetic diversity across seven vegetation zones. We also measured phylogenetic structure using the net relatedness index (NRI) and the nearest species index (NTI). Our results show that lower montane wet forest has the highest level of species richness, density, and phylogenetic diversity of woody plants, while lower montane dry forest has the highest level of species richness, density, and phylogenetic diversity in herbaceous plants. In total plants, NRI and NTI of four forest zones were smaller than three alpine zones. In woody plants, lower montane wet forest and upper montane forest have overdispersed phylogenetic structures. In herbaceous plants, NRI of Afro‐alpine zone and nival zone are smaller than those of bamboo zone, upper montane forest, and heath zone. We suggest that compared to open dry forest, humid forest has fewer herbaceous plants because of the closed canopy of woody plants. Woody plants may have climate‐dominated niches, whereas herbaceous plants may have edaphic and microhabitat‐dominated niches. We also proposed lower and upper montane forests with high species richness or overdispersed phylogenetic structures as the priority areas in conservation of Mount Kenya and other high mountains in the Eastern Afro‐montane biodiversity hotspot regions.

## 1 INTRODUCTION

Plant richness in high mountains is one of the most important issues in biodiversity conservation, due to global climate change (Bhattarai & Vetaas, 2003; Li, Kraft, Yu, & Li, 2015; Trigas, Panitsa, & Tsiftsis, 2013; Vetaas & Grytnes, 2002). It is well known that species richness and endemism change along environmental gradients, but the associated patterns in the variation of genetic and evolutionary diversity have not been adequately addressed. Phylogenetic diversity (PD) (i.e., the sum total of branch lengths of the phylogeny linking the species in an area; Faith, 1992) is a biodiversity index which quantifies the combined phenotypic or genetic diversity across the species (Cadotte & Davies, 2016; Davies et al., 2007; Purvis & Hector, 2000). Phylogenetic diversity reflects the underlying evolutionary history represented by a set of taxa, and it has become a subject of interest to both ecologists seeking to understand the influence of evolutionary history on species abundance and interactions, and to conservation biologists wishing to prioritize evolutionary history for conservation purposes (Forest et al., 2007; Li et al., 2015; Sechrest et al., 2002; Tucker et al., 2017). There is a positive relationship between phylogenetic diversity and species richness (Kluge & Kessler, 2011; Sax et al., 2007), especially along an elevational gradient where changes in species richness are apparent (Li et al., 2015).

The relative similarity of species and phylogenetic diversity across space is dependent on the underlying environmental conditions and biogeographical history. It could be that species richness and phylogenetic diversity are highly congruent due to random or even selection of taxa across a phylogeny (Rodrigues, Brooks, & Gaston, 2005). Conversely, highly nonrandom patterns, for example, phylogenetic signals in environmental tolerances or localized speciation events, could create incongruent taxonomic and phylogenetic diversity patterns (Devictor et al., 2010; Tucker & Cadotte, 2013). Given these complexities, both taxonomic and phylogenetic diversity should be evaluated for conservation. For example, Li et al. (2015) found that compared to other communities, the evergreen broad‐leaved forests in Dulong Valley in China had the highest levels of species richness and phylogenetic diversity, as well as an overdispersed phylogenetic structure, and suggest that communities with high species richness or an overdispersed phylogenetic signal should be the focus for biodiversity conservation, as these areas may help maximize the potential of local flora to respond to future global change.

When evaluating diversity patterns, it is also important to compare and contrast species belonging to different ecological guilds. This is due to the fact that differences in life history traits or mechanisms of resource uptake can lead to highly disparate responses to outside inputs such as climate. For plants, the distinction between woody and herbaceous growth forms is probably the most profound contrast in terrestrial ecosystems (FitzJohn et al., 2014). Herbaceous and woody taxa are believed to be differentially influenced by environmental factors such as precipitation and temperature (Whittaker, 1965), which are nonrandomly associated with the evolutionary history of these taxa and their traits (Díaz et al., 2016). Thus, it may be that factors resulting in variation in species richness and phylogenetic diversity might differ between woody and herbaceous plants, and comparisons between these communities may provide better insight into the factors influencing the distribution of total plant biodiversity (Bhattarai & Vetaas, 2003).

Here, we analyze changes in taxonomic and phylogenetic diversity across different vegetation zones of Mount Kenya, which is the largest ancient extinct volcano in the Great Rift Valley area, and the second highest peak in Africa (Speck, 1982). It constitutes an important reservoir for plant diversity, including a substantial number of endemic and endangered species. As the publishing of the first checklist of about 140 plant species by Hooker and Oliver in 1885, numerous studies have studied plant and vegetation diversity in Mount Kenya (e.g., Bussmann, 1994; Fries & Fries, 1948; Niemelä & Pellikka, 2004; Young & Peacock, 1985). However, plant taxonomic and phylogenetic diversity of Mount Kenya have not been thoroughly analyzed, and we lack critical knowledge on the evolutionary dimension of the biodiversity in this region.

The aim of this study was to quantify the relationship between species richness and phylogenetic diversity and to explore community phylogenetic structure across vegetation zones of Mount Kenya, Kenya. We also aim to compare the diversity patterns between woody and herbaceous plants in order to a get better insight into the factors contributing to observed patterns in diversity.

## 2 MATERIALS AND METHODS

### 2.1 Study area

**Figure 1:**
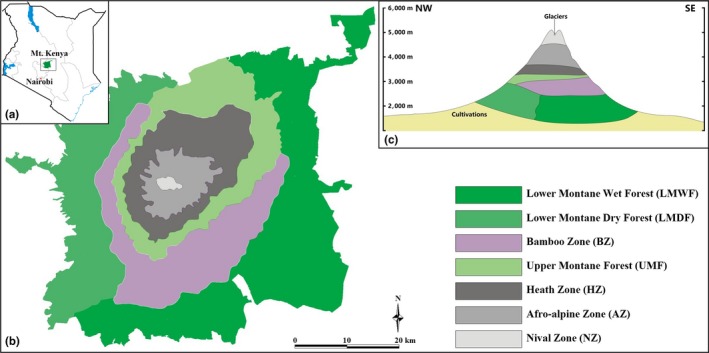
Vegetation zones of Mount Kenya. (a) The location of Mount Kenya in Kenya. (b) The vegetation zones of Mount Kenya in top view (adapted from Niemelä & Pellikka, 2004). (c) The vegetation zones of Mount Kenya from northwest to southeast in lateral view (adapted from Coe, 1967 and VECEA Team, 2012) [Colour figure can be viewed at http://wileyonlinelibrary.com]

**Figure 2:**
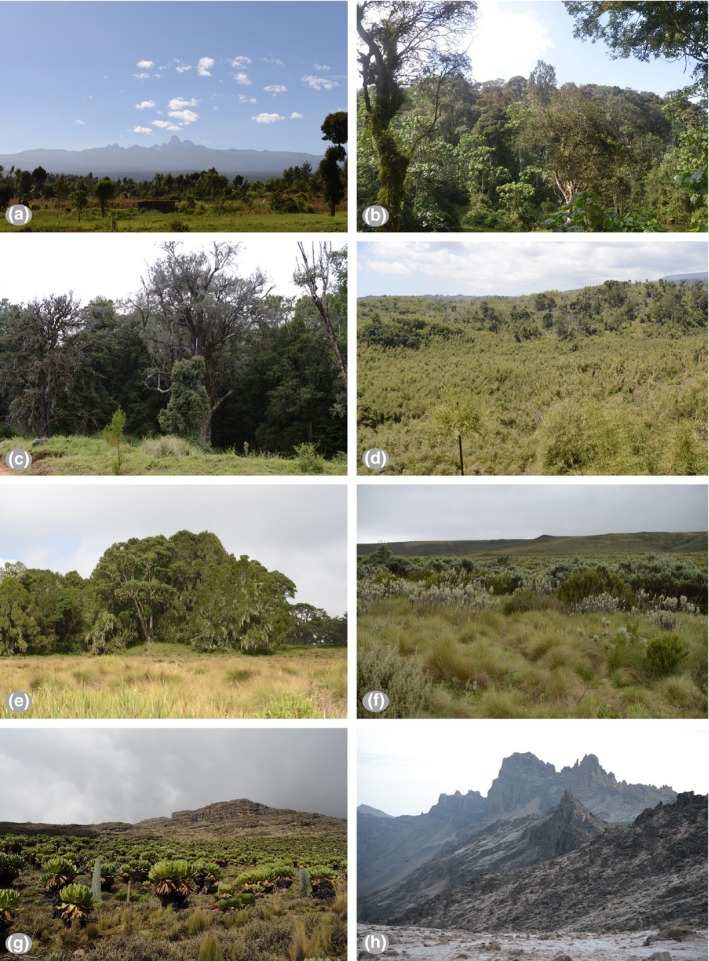
Vegetation zones of Mount Kenya. (a) Distant overview of Mount Kenya; (b) lower montane wet forest (LMWF); (c) lower montane dry forest (LMDF); (d) bamboo zone (BZ); (e) upper montane forest (UMF); (f) heath zone (HZ); (g) Afro‐alpine zone (AZ); and (h) nival zone (NZ). Photograph was taken by G.W. Hu (a, c, d, e, f, g and h) and Y.D. Zhou (b) [Colour figure can be viewed at http://wileyonlinelibrary.com]

Mount Kenya is located in central Kenya, approximately 193 km northeast of Nairobi and 480 km from Kenyan coast (0°10′S, 37°20′E; Figure 1). Elevation ranges from a low of 1,500 m asl to a high of 5,192 m asl at the tallest peak. Along this elevation gradient, annual precipitation varies from a low of about 870 mm at the base of the mountain to about 1,970 mm at the peak and temperature from about 12°C to about −4°C. This steep climate gradient results in a dramatic change in vegetation cover. The lower slopes are composed primarily of montane forest, with some big trees being dominant. Above the forest, there are large tracts of bamboo forest, particularly on the east and southeast slopes. The upper montane forest is dominated by *Podocarpus* spp. and *Hagenia abyssinica* trees, after which a dominant heath zone is present. Above heath zone the vegetation becomes dominated by alpine specialists such as *Dendrosenecio* spp. and *Lobelia* spp. Above 4,500 m, asl is largely unvegetated and partially glaciated. We classified these changes in vegetation into seven primary zones: lower montane wet forest (LMWF), lower montane dry forest (LMDF), bamboo zone (BZ), upper montane forest (UMF), heath zone (HZ), Afro‐alpine zone (AZ) and the nival zone (NZ) (Figures 1 and 2; Table 1) (Coe, 1967; Niemelä & Pellikka, 2004; VECEA team, 2012).

**Table 1:** Elevation ranges, area, annual mean precipitation, and representative species for the seven vegetation zones of Mount Kenya

Zones	Elevation (m)	Area (km^2^)	Annual mean precipitation/mm	Representative species
Lower Montane Wet Forest (LMWF)	1,450–3,110	852	1,090–2,170	*Newtonia buchananii* (Baker) G.C.C. Gilbert & Boutique Ocotea usambarensis Engl. Tabernaemontana stapfiana Britten Vitex keniensis Turrill Xymalos monospora (Harv.) Baill. ex Warb. Zanthoxylum gilletii (De Wild.) P.G. Waterman
Lower Montane Dry Forest (LMDF)	1,850–3,030	456	870–1750	*Juniperus procera* Hochst. ex Endl. *Olea europaea* L.
Bamboo Zone (BZ)	2,140–3,270	400	1,260–1,910	*Lobelia bambuseti* R.E. Fr. & T.C.E. Fr. Sambucus africana Standl. *Yushania alpina* (K. Schum.) W.C. Lin
Upper Montane Forest (UMF)	2,650–3,890	342	1,310–1,800	*Hagenia abyssinica* J.F. Gmel. *Hypericum revolutum* Vahl *Podocarpus latifolius* (Thunb.) R. Br. ex Mirb.
Heath Zone (HZ)	3,040–4,240	270	1,540–1,860	Alchemilla argyrophylla Oliv. Erica arborea L. *E. trimera* subsp. *keniensis* (S. Moore) Beentje *Protea caffra* subsp. *kilimandscharica* (Engl.) Chisumpa & Brummitt
Afro‐alpine Zone (AZ)	3,380–4,790	119	1,720–1,970	*Carduus keniensis* R.E. Fr. Dendrosenecio keniodendron (R.E. Fr. & T.C.E. Fr.) B. Nord. *D. keniensis* (Baker f.) Mabb. *Festuca pilgeri* St.‐Yves *Lobelia gregoriana* Baker f. *L. telekii* Schweinf.
Nival Zone (NZ)	4,500–5,199	9	1,860–1,970	*Arabis alpina* L. Helichrysum brownei S. Moore *Senecio purtschelleri* Engl. *Valeriana kilimandscharica* Engl.

### 2.2 Data sources

We compiled a comprehensive checklist of seed plants based on data from various scientific expeditions to Mount Kenya since the 1900s. These data sources include published floras and field guides such as *Flora of Tropical East Africa* (FTEA editors, 1952), *Upland Kenya Wild Flowers and Ferns* (Agnew, 2013), *Wild Flowers of East Africa* (Blundell, 1987) and *Kenya Trees Shrubs and Lianas* (Beentje, 1994), data of specimens from East African Herbarium, Nairobi, Kenya (EA) and Global Biodiversity Information Facility (GBIF, https://www.gbif.org/), and data from our own scientific expedition from 2009 to 2016 (specimens were stored at Herbarium of Wuhan Botanical Garden, Wuhan, China, HIB). Species were assigned to vegetation zones according to the sampling locations and habitat descriptions described in the monographs. Growth forms of species were classified as either woody or herbaceous plants.

### 2.3 Taxonomic metrics

To eliminate the area effect on species richness in zones of different sizes, species density (D) for each zone was calculated based on the following equation (Li et al., 2015; Vetaas & Grytnes, 2002):

D=S/ln(A)

where *S* is number of species in each zone and *A* is the area of each zone.

### 2.4 Phylogeny construction

Our primary goal was to calculate phylogenetic distance metrics and we first constructed a phylogenetic tree for all the seed plants of Mount Kenya using the Phylomatic program with the stored tree from Zanne et al. (2014) (Webb & Donoghue, 2005). The branch lengths of the supertree, in millions of years, were used to create a distance matrix with cophenetic distances. We also built two phylogenetic trees using only woody and herbaceous plants, respectively.

### 2.5 Phylogeny metrics

We calculate two diversity measures PD and SES_PD, for total, woody, and herbaceous plants of each zones. Faith's phylogenetic diversity (PD) is widely used in several conservation studies, although it is positively correlated with species richness (Li et al., 2015). A null model to standardize PD measurements and standard effect size phylogenetic diversity (SES_PD) was also calculated by dividing the difference between the observed and expected PD by the standard deviation of the null distribution. Phylogenetic structure was calculated using the net relatedness index (NRI) and the nearest species index (NTI) (Webb, Ackerly, McPeek, & Donoghue, 2002) as follow:

(1) $$\text{NRI}=-1\times \left({\text{MPD}}_{\mathrm{observed}}-{\text{MPD}}_{\mathrm{randomized}}\right)/{\text{sdMPD}}_{\mathrm{randomized}}$$

(2) $$\text{NTI}=-1\times \left({\text{MNTD}}_{\mathrm{observed}}-{\text{MNTD}}_{\mathrm{randomized}}\right)/{\text{sdMNTD}}_{\mathrm{randomized}}$$

MPD refers to the average phylogenetic relatedness between all possible pairs of taxa in an assemblage. MPD_observed_ is the observed MPD, MPD_randomized_ is the expected MPD of randomized assemblages, and sdMPD_randomized_ is the standard deviation of randomized MPD. Randomization involved shuffling the species identities of individuals in the sample. MNTD represents the mean phylogenetic relatedness between each species and its nearest relative in the assemblage. MNTD_observed_ is the observed MNTD, MNTD_randomized_ is the expected MNTD of randomized assemblages, and sdMNTD_randomized_ is the standard deviation of randomized MNTD. We used 999 randomized communities for each analysis to assess the statistical significance of the observed patterns. A two‐tailed significance test was used to assess whether these NRI/NTI results differed significantly from zero. Consequently, positive NRI/NTI values indicate phylogenetic clustering that species are more closely related than expected, and negative NRI/NTI values indicate phylogenetic overdispersion and species in communities are more distantly related than expected (Webb et al., 2002). All the phylogenetic analyses were performed in R 3.3.3 software (R Core Team, 2017) with the picante package (Kembel et al., 2010).

## 3 RESULTS

### 3.1 Species richness, density, and phylogenetic diversity of seed plants

**Figure 3:**
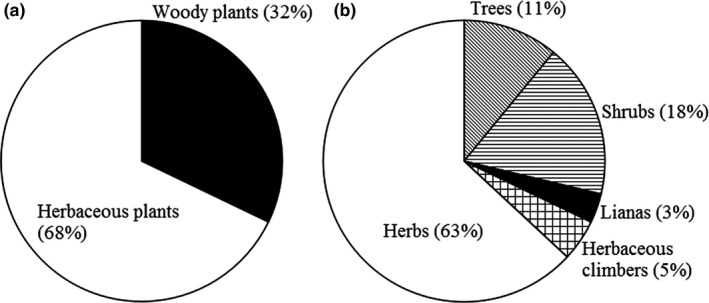
Species richness of woody and herbaceous plants in Mount Kenya. (a) Species richness of woody and herbaceous plants; (b) species richness of trees, shrubs, lianas, herbaceous climbers, and herbs

A total of 1335 seed plants of Mount Kenya were compiled across all of the vegetation surveys, including subspecies and varieties, which belonged to 628 genera and 134 families. There were 429 woody plants (32% in total, including 146 trees, 238 shrubs, and 45 lianas) and 906 herbaceous plants (68% in total, including 64 herbaceous climbers and 842 herbs) (Figure 3). As expected, species richness, density, and phylogenetic diversity were found to be the highest in low montane dry forest (LMDF) and the lowest in nivial zone (NZ), while the SES_PD was found to be the highest in low montane wet forest (LMWF) and the lowest in Afro‐alpine zone (AZ) (Table 2).

**Table 2:** Species richness (SR), density (D), phylogenetic diversity (PD), and standard effect size phylogenetic diversity (SES_PD) of total, woody, and herbaceous plants among different vegetation zones of Mount Kenya

Zones	Total plants				Woody plants				Herbaceous plants			
	SR	*D*	PD	SES_PD	SR	*D*	PD	SES_PD	SR	*D*	PD	SES_PD
LMWF	832	123.30	39,230.48	2.15	327	48.46	17,930.22	0.65	505	74.84	23,737.65	1.37
LMDF	905	147.82	39,279.75	−3.30	271	44.26	15,077.24	−1.12	634	103.55	26,603.27	−2.99
BZ	354	59.08	17,855.20	−3.29	71	11.85	5,232.94	−0.40	283	47.23	13,887.90	−2.31
UMF	283	48.50	14,921.75	−2.78	50	8.57	4,144.94	0.29	233	39.93	11,802.95	−2.42
HZ	322	57.52	15,332.14	−5.41	46	8.22	3,286.01	−1.66	276	49.30	12,919.84	−4.04
AZ	192	40.17	9,504.18	−5.49	23	4.81	1,722.95	−1.84	169	35.36	8,337.71	−4.37
NZ	65	29.58	3,689.96	−4.52	7	3.19	666.38	−0.80	58	26.40	3344.03	−3.81

There were notable differences between the diversity of herbaceous and woody communities. SR, *D*, and PD of woody plants were highest in LMWF, while those of herbaceous plants were highest in LMDF. The SES_PD of woody and herbaceous plants was both highest in LMWF and LMDF. The lowest SR, *D*, and PD were found in NZ, for both woody and herbaceous plants, while the lowest SES_PD was both found in AZ (Table 2).

### 3.2 Phylogenetic structure of total, woody, and herbaceous plants

**Figure 4:**
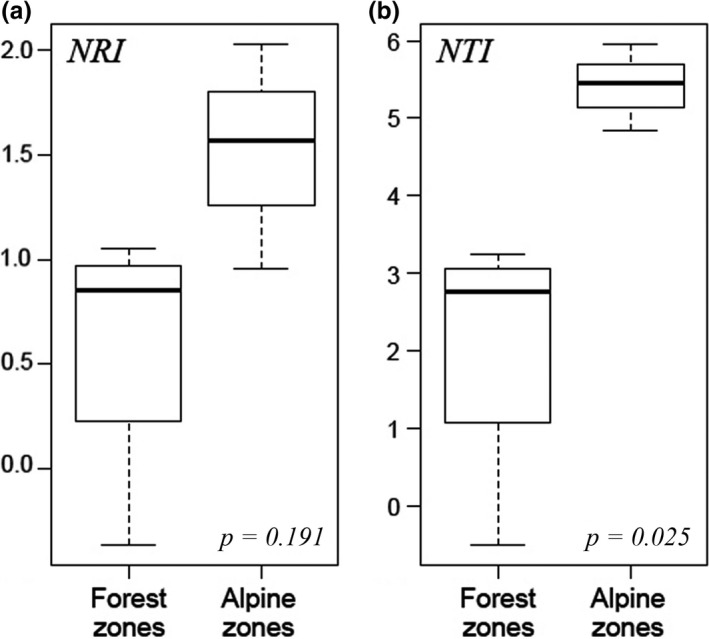
Comparison of (a) NRI and (b) NTI between forest zones (LMWF, LMDF, BZ, and UMF) and alpine zones (HZ, AZ, and NZ). Solid black lines in the central of boxes represent quantile values. *P*‐values show the significance *t* tests

**Figure 5:**
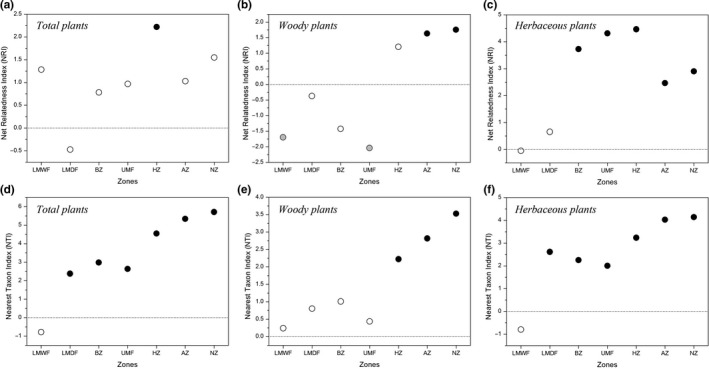
Variation in community phylogenetic relatedness among zones of Mount Kenya. Positive index values indicate phylogenetic clustering, and negative values indicate phylogenetic overdispersion. Values with *P *≤* *0.05 are depicted as black points, values with 0.05 > *P *>* *0.95 are depicted as white points, and values with *P* ≥ 0.95 are depicted as gray points

When we examined differences between forest zones (LMWF, LMDF, BZ, and UMF) and alpine zones (HZ, AZ, and NZ), both NRI and NTI showed substantially increased pattern while NRI is not significant (*P *=* *0.191) and NTI is significant (*P *<* *0.05; Figure 4). NRI and NTI showed different patterns in total, woody, and herbaceous plants (Figure 5). For total plants, NRI of LMDF and NTI of LMWF were negative, indicating phylogenetic overdispersion in these two zones. In contrast, NRI and NTI of other zones were positive, indicating phylogenetic clustering (Figure 5a,d). For woody plants, all the NTI were positive, while NRI in LMWF, LMDF, BZ, and UMF were negative, indicating phylogenetic overdispersion in these four zones (Figure 5b,e). For herbaceous plants, most of NRI and NTI values were positive, indicating phylogenetic clustering, while NRI and NTI of LMWF were nonsignificant negative values (Figure 5c,f).

## 4 DISCUSSION

The species richness of woody and herbaceous plants of forests is expected to be affected by environmental variation, forest canopy, and anthropogenic disturbances (Clinton, 2003; Schmitt, Denich, Demissew, Friis, & Boehmer, 2010; Zhang, Huang, Wang, Liu, & Du, 2016). Woody plants are probably more strongly influenced by large‐scale environmental variations than herbaceous plants, such as the changes of moisture, temperature, or altitude (Schmitt et al., 2010). That is to say, the difference in humidity between the southeast and northwest slopes of Mount Kenya has a stronger influence on species richness of woody plants, and lead the LMWF to have the highest level of species richness of woody plants. The density of forest canopy could influence the light intensity, moisture, and temperature in the understory (Clinton, 2003; Whittaker, 1960). There are many big trees found in LMWF, such as *Newtonia buchananii* (Fabaceae), *Ocotea usambarensis* (Lauraceae), *Vitex keniensis* (Lamiaceae), *Tabernaemontana stapfiana* (Rubiaceae), and *Zanthoxylum gilletii* (Rutaceae) (Table 1). These tall trees as well as other small trees, shrubs, or lianas form highest level of species density of wood plants, which blocks sunshine, leading to stunted development of understory herbaceous plants (Table 2). In contrast, LMDF is dominated by small trees and shrubs, as well as few large trees, such as *Juniperus procera* (Cupressaceae) and *Olea europaea* (Oleaceae), and this leads to better development of understory herbaceous plants due to light availability. In addition, the areas with moderate impacts may have greater environmental heterogeneity, which could provide greater opportunities for plants to growth (Pausas & Austin, 2001; Zhang et al., 2016). The degree of human disturbance on the northwest slope of Mount Kenya is obviously greater than that in the southeast slope, for example, the two famous mountaineering routes of Mount Kenya, Naromoru Track and Sirimon Track, are both located in the west slope of this mountain, which with more tourist activities every year. This could lead the LMDF with moderate impacts has the highest level of total species richness.

Although NRI and NIT of LMWF have no significant negative values, most of these two indices showed positive values in different zones of Mount Kenya and had a generally increasing trend as the zones changed, and this indicates that herbaceous plants in Mount Kenya have clustering phylogenetic structures, and tend to be more phylogenetically clustered at higher elevations (Figure 5e,f). Interestingly, the NRI of herbaceous plants of AZ and NZ was smaller than those of BZ, UMF, and HZ, and a similar pattern could be found in NRI of total plants (Figure 5). This phenomenon was also found in areas above 5,500 m asl. in Hengduan Mountains, China, and this can be attributed to the fact that in the upper elevation zones, the plants were sparsely distributed and this lead to a decrease in interspecific competition (Li, Zhu, Niu, & Sun, 2013). While, in woody plants, there are obvious increasing trends of NRI and NTI as the zones transition from UMF to NZ (Figure 5). Combining the phylogenetic structure patterns of woody and herbaceous plants, we strongly support that, large woody plants have climate‐dominated niches, whereas herbaceous plants have edaphic and microhabitat‐dominated niches, and the temperature or climate filtering process presumably has played a greater role in structuring species into local communities for woody plants than for herbaceous plants (Qian, Jin, & Ricklefs, 2017; Ricklefs & Latham, 1992).

The phylogenetic structure (NRI and NTI) of total plants cluster further as the vegetation change from forest to alpine zones (Figure 4), and this result supports that, competition structured communities at low elevation areas, while at the high elevation areas, the environmental stress acted as a filter on lineages due to lower temperatures and unstable climate (Li et al., 2013; Machac, Janda, Dunn, & Sanders, 2011; Webb et al., 2002). Our results also showed that phylogenetic structures have different patterns for different growth forms as the vegetation zones changed. For woody plants, NRI and NTI increase as the vegetation zones change with a minor peak both in NRI and in NTI at the upper montane forest (UMF). A similar phenomenon was reported in central Veracruz, Mexico, with a sharp drop of NRI at around 2500 m asl, which is a representative of Cloud forest (Gómez‐Hernández et al., 2016). In Mount Kenya, the upper montane forest is also called Cloud forest or Moist forest, although rainfall in this region is lower than in the montane rainforest zone, evaporation is also lower, and frequent heavy mists contribute to the humidity (Niemelä & Pellikka, 2004). It has different characteristics and includes tree species such as *Hagenia abyssinica* (Rosaceae), *Hypericum revolutum* (Hypericaceae), and *Juniperus procera* (Cupressaceae) (Lange et al., 1997; Niemelä & Pellikka, 2004). Our result of phylogenetic structures of woody plants suggested the most phylogenetic overdispersed zone in Mount Kenya occurred at the ecotone where different zones overlapped with equal chances of each dominating (Gómez‐Hernández et al., 2016).

Recently, several studies explicitly compared biodiversity conservation prioritizations based on both species richness and phylogenetic diversity criteria to assess the efficacy of each approach (Forest et al., 2007; Kraft, Baldwin, & Ackerly, 2010; Li et al., 2015; Vandergast, Bohonak, Hathaway, Boys, & Fisher, 2008). These authors suggested that biodiversity conservation is maximized by the inclusion of communities and zones with overdispersed phylogenetic structure, because such natural areas include phylogenetically distantly related lineages (Li et al., 2015). Other have suggested that proportional endemism is a more important consideration than phylogenetic diversity in developing conservation priorities (Brewer, 2017), but we were not able to assess proportional endemism of each vegetation of Mount Kenya in the current study. In this case, the two lower montane forest zones (LMWF and LMDF) and the upper montane forest (UMF) of Mount Kenya should be given as much attention in conservation as the alpine zones because these forest zones are the most phylogenetically diverse and also have the highest species richness which is the key to the maintenance of biodiversity (Vanleeuve et al., 2003). The mountain chains of eastern Africa, which extend from the Ethiopian mountains, through east African nations and southwards to Mozambique, are formed as the result of an active continental rift (Chorowicz, 2005). It contains the Eastern Afromontane biodiversity hotspot (EABH), which is the one out of eight biodiversity hotspots of the Afromadagascan region (Mittermeier, Turner, Larsen, Brooks, & Gascon, 2011). The kind of degradation experienced in Mount Kenya is similar to that found in the lower montane forests of most other mountains in EABH (Giliba et al., 2011; Lambrechts et al., 2003; Lambrechts, Woodley, Hemp, Hemp, & Nnyiti, 2002; Petursson, Vedeld, & Sassen, 2013; Teketay, 1992). Our findings have a profound effect on biodiversity conservation across the EABH as the vegetation types in these mountains are similar to those of Mount Kenya (Bussmann, 2006). It is important that the lower and upper montane forests of EABH should be given as much attention in conservation just like Mount Kenya, for they also have the highest species richness and most diverse phylogenetic lineages, in addition to having the highest evolutionary potential (Faith, 1992; Forest et al., 2007; Li et al., 2015).

## CONFLICT OF INTEREST

None declared.

## AUTHOR CONTRIBUTION

Y.Z. and S.C. conceived and wrote the manuscript. Y.Z. and G.H. provided and analyzed the data. G.M., X.Y., and Q.W. provided the idea. All authors reviewed the manuscript.

## DATA ACCESSIBILITY

Data are available via the Dryad Digital Repository: https://doi.org/10.5061/dryad.7sj4t8h.
